# Extraction and Recovery of Flavonoids from Tartary Buckwheat Using Deep Eutectic Solvents

**DOI:** 10.3390/molecules31081261

**Published:** 2026-04-11

**Authors:** Xueting Feng, Tingting Huang, Jinmei Feng, Xiaoling Wang

**Affiliations:** College of Pharmacy and Food, Southwest Minzu University, Chengdu 610041, China; huangtt0404@163.com (T.H.); 19169384283@163.com (J.F.)

**Keywords:** deep eutectic solvents, flavonoids, Tartary buckwheat, extraction, recovery

## Abstract

In recent years, the green extraction of natural active ingredients has generated widespread attention. And deep eutectic solvents have widely replaced traditional organic solvents. In this study, choline chloride/glycolic acid (1:2) was chosen as the optimal extractant to extract flavonoids from Tartary buckwheat. The optimal extraction conditions were as follows: water content of 30%, liquid–solid ratio of 40 mL/g, extraction temperature of 60 °C and extraction time of 40 min. And the extraction efficiency reached 27.22 ± 0.31 mg/g. Then kinetic and thermodynamic mechanisms were investigated comprehensively, and the results showed that the extraction process could be well fitted by Fick’s second law. In addition, macroporous resins were used to recover flavonoids from extracts. The adsorption efficiency of flavonoids on HP20 resins under the optimal conditions (time of 2 h, liquid–resin ratio of 2.5 mL/g, temperature of 25 °C) could reach 80.14 ± 0.33%.

## 1. Introduction

**Figure 1:**
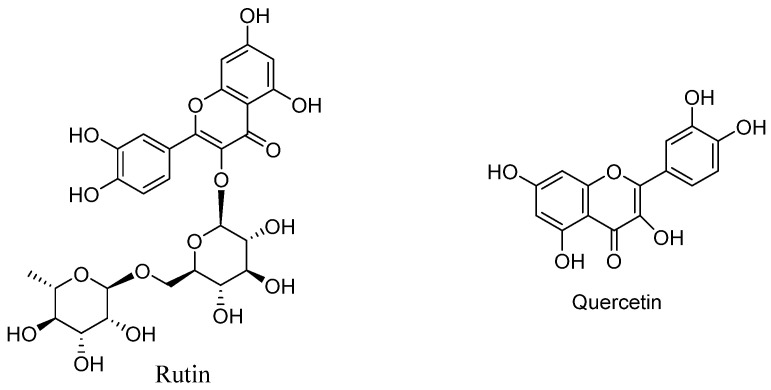
Chemical structures of rutin and quercetin.

Tartary buckwheat (*Fagopyrum tataricum* Gaertn., TB) has been known as the “king of grains” since ancient times, with functions such as lowering blood pressure, blood sugar, blood lipids, and antioxidants [1,2]. It is often prepared into buckwheat tea through some primary processing, which has certain health functions. As a dual-use crop for medicine and food, TB contains multiple bioactive ingredients, especially flavonoids, whose main components are rutin and quercetin (Figure 1) [3]. TB is widely distributed in high-altitude areas of Asia, Europe and North America, but the utilization of it is mainly based on primary processing, such as preparing Tartary buckwheat tea. There is increasing attention paid to the health benefits of natural products such as flavonoids, and a large amount of flavonoids in TB; most of them have certain pharmacological activities, such as rutin, which has a wide range of pharmacological activities (e.g., antioxidant, anti-inflammatory and even anti-SARS-CoV-2) [4,5]. Therefore, it is necessary to carry out deep processing, for example, in the extraction of flavonoids.

The extraction solvent plays a significant role in influencing the extraction efficiency [6]. Therefore, an appropriate solvent should be selected to extract the target compounds. For the extraction of flavonoids, the traditional method is generally solvent extraction, and commonly used extraction solvents include methanol, ethanol, and ethyl acetate [7,8,9]. Cao et al. [10] conducted the ultrasonic-assisted extraction of flavonoids from *Triarrhena lutarioriparia* using 70% ethanol under a liquid-to-solid ratio of 40 mL/g for 60 min, achieving an extraction rate of 15.88 mg/g. Lai et al. [11] used different solvents (95% ethanol, 70% aqueous, methanol, and ethyl acetate) to extract flavonoids from navel orange peel. The results showed that ethyl acetate was the best solvent for the extraction. This may be due to the more similar polarity between ethyl acetate and the main flavonoids in orange peel, such as hesperidin and narirutin, which is consistent with the principle of “similar dissolving”. Despite the favorable extraction efficiency achieved by these organic solvents, their disadvantages cannot be ignored. For example, they are known to be volatile, and harmful to both the environment and human health. With increasing attention to environmental and resource issues, green solvent extraction technology has gradually become a research hotspot in natural product extraction [12].

Deep eutectic solvents (DESs), considered as a new generation of green solvents, are obtained by simply mixing hydrogen bond acceptors (HBAs) and hydrogen bond donors (HBDs); their physicochemical properties have many similarities with ILs, such as low vapor pressure, and can increase the solubility of target compounds by selecting different HBAs and HBDs [13]. In addition, due to the fact that DESs are mainly composed of non-toxic, relatively safe and cheap compounds, and the preparation process is simple and the product purity is high, they are good substitutes for traditional organic solvents and even ionic liquids [14], and have been widely used in the development of green extraction and separation methods [15,16,17]. Luo et al. [18] compared the extraction effects of traditional organic solvents and 17 DESs on flavonoids from Sophorae Tonkinensis Radix Et Rhizoma. The results showed that choline chloride/acrylic acid (1:4) was the optimal extraction solvent, due to the suitable viscosity as well as its similar polarity to flavonoids. And under the conditions of water content of 20%, solid–liquid ratio of 1:148 g/mL, extraction temperature of 65 °C and extraction time of 61 min, the flavonoid extraction efficiency was 3.785 ± 0.026 mg/g, which was higher than that of 80% methanol and 80% ethanol extracts. Peng et al. [19] also extracted the flavonoids from Moringa oleifera Lam. leaves, and compared the extraction efficiency between DESs and traditional solvents (70% ethanol). The results showed that the extraction rate of DESs composed of betaine and urea (molar ratio of 1:2) was 1.7 times higher than that of 70% ethanol under the same conditions. Therefore, compared with traditional solvent extraction, the extraction with DESs is greener, more environmentally friendly, and more efficient.

This work aimed to establish the environmentally friendly extraction of flavonoids from TB using the DES. The types of DESs and the extraction conditions were studied by performing single-factor experiments. Then the kinetic and thermodynamic mechanisms were investigated. Furthermore, macroporous resins were used to recover flavonoids from DES extracts. The procedure developed in this study will provide a foundation for the extraction of flavonoids from TB.

## 2. Results and Discussion

### 2.1. Investigation on Extraction Conditions

#### 2.1.1. Effect of the DES Types on the Extraction Efficiency

**Figure 2:**
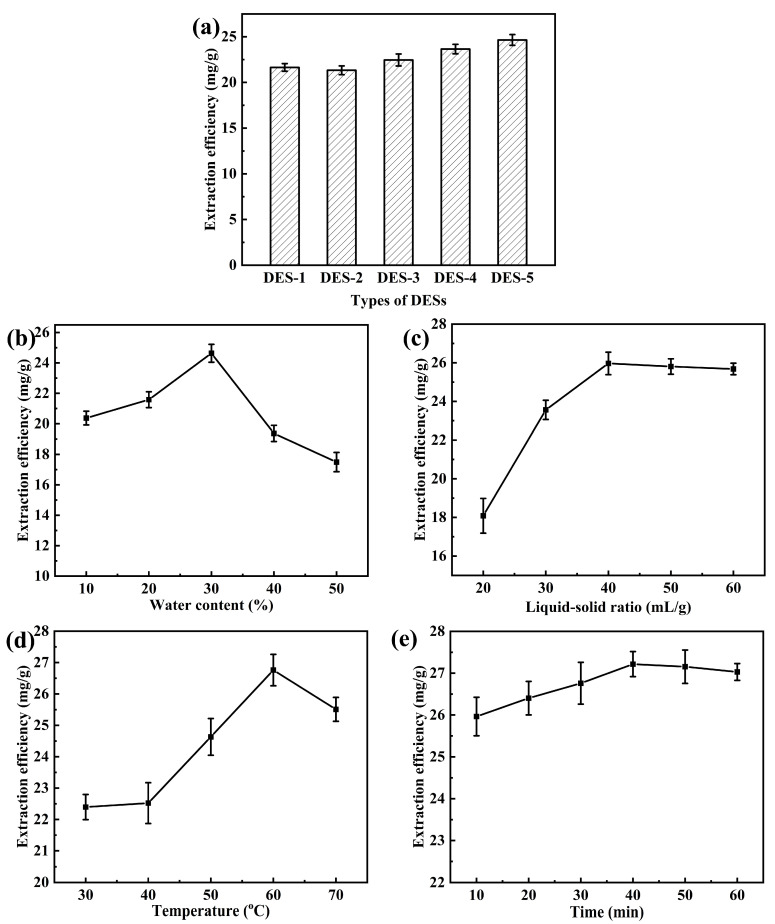
Effect of the (**a**) DES types, (**b**) water content, (**c**) liquid–solid ratio, (**d**) temperature and (**e**) time on the extraction efficiency of flavonoids.

The physical and chemical properties of DESs can be greatly affected by the combination of HBAs and HBDs, so their extraction performance for different compounds is very different. The choice of a suitable DES is an essential stage in the extraction process, since it has significant impacts on the extraction efficiency [20]. So, in this study, five DESs (DES-1 to DES-5) were prepared and screened to effectively extract flavonoids from TB. In detail, the water content of the extraction solvent was 30%, and the extraction was carried out at 50 °C for 30 min with a liquid–solid ratio of 40 mL/g. The results are shown in Figure 2a.

From Figure 2a, it can be seen that different DESs had different extraction efficiencies, and DES-5 showed the highest extraction efficiency, followed by DES-4, DES-3, DES-1 and DES-2. This may be due to the fact that the glycolic acid in DES-5 has two hydroxyl groups and one carbonyl group, which can form hydrogen bonds with the carbonyl and hydroxyl groups of flavonoid structural species, thus exhibiting good extraction efficiency [21]. However, glycerol in DES-1 and 1,3-propanediol in DES-2 only contain hydroxyl groups, and the hydrogen bonds formed with flavonoids are slightly weaker, resulting in lower extraction efficiency. Therefore, DES-5 (choline chloride/glycolic acid, 1:2) was chosen for further experiments.

#### 2.1.2. Effect of the Water Content on the Extraction Efficiency

A major challenge for a DES as an extraction solvent is its high viscosity, which is not conducive to the diffusion of the target compounds. And the issue can be solved by adding the proper amount water, because the addition of water can change the system’s viscosity and polarity, which can significantly affect the extraction and distribution rates [22]. In this study, the different water contents (10–50%, *v*/*v*) were selected to investigate their effects on the extraction efficiency. Other extraction conditions were kept constant as follows: a liquid–solid ratio of 40 mL/g, extraction time of 30 min and extraction temperature of 50 °C.

According to the results shown in Figure 2b, the extraction efficiency was increased with an increase in water content from 10% to 30%. The addition of water decreased the viscosity of the extraction solvent, and increased the mass transfer, so the extraction efficiency was increased. However, with a further increase in water content from 30% to 40%, a decrease in the extraction efficiency was observed. This may be due to the core of DES-enhanced extraction involving hydrogen bonding, and the addition of excessive water (>30%) could break the hydrogen bonds between the DES and flavonoids [23]. This caused a decrease in the solubility of flavonoids in the extraction solution; therefore, the extraction efficiency decreased. Thus, 30% was chosen as the optimum water content for subsequent experiments.

#### 2.1.3. Effect of Liquid–Solid Ratio on the Extraction Efficiency

The liquid–solid ratio is an important factor that affects the extraction efficiency as well as the cost. When the amount of solvent extracted increases, the contact and interaction between the solvent and sample will be enhanced, which is beneficial for improving the extraction efficiency until the extraction equilibrium is reached. However, too large amounts of solvent will increase the difficulty and cost of the subsequent concentration together with recovery processes [24]. Therefore, the effect of the liquid–solid ratio (20, 30, 40, 50 and 60 mL/g) on the extraction efficiency was investigated, under the conditions of water content 30%, extraction time 30 min, and extraction temperature 50 °C. The results are shown in Figure 2c. It was found that the extraction efficiency increased at first and then slightly decreased with an increase in the liquid–solid ratio. When the liquid–solid ratio was too low, the extraction solvent could not fully contact the TB powder, resulting in a low extraction efficiency. However, a high ratio could dissolve more impurities, which competed with the flavonoids for the solvent or altered the solvent’s properties, thereby reducing the solubility of the flavonoids [25]. Therefore, 40 mL/g was determined to be the optimum liquid–solid ratio since it produced the maximum extraction efficiency.

#### 2.1.4. Effect of Temperature on the Extraction Efficiency

An increase in temperature can reduce the viscosity of DESs, enhance the diffusion of substances in the solvent, and also increase the solubility of substances in the solvent; therefore, the temperature rise is beneficial for improving extraction efficiency. However, higher temperatures raise the cost of the extraction and may damage the flavonoids [26], so in this study, the effect of temperature was assessed by experimenting with five temperature points (30 °C, 40 °C, 50 °C, 60 °C and 70 °C), and the other conditions were kept constant: water content 30%, extraction time 30 min, and liquid–solid ratio 40 mL/g. The results are shown in Figure 2d. It can be seen that the extraction efficiency increased as the temperature increased from 30 °C to 60 °C, but a decrease was observed when the temperature was higher than 60 °C. As the temperature increased, the viscosity of the DES decreased, molecular motion accelerated, and the mass transfer resistance of the target compound decreased, resulting in relatively high extraction efficiency. However, an excessively high temperature could cause structural damage or degradation of flavonoids, resulting in a decrease in extraction efficiency [27]. So, 60 °C was selected as the optimum temperature for the extraction.

#### 2.1.5. Effect of Time on the Extraction Efficiency

Extraction time is another key factor that affects extraction efficiency. If the time is too short, it may result in insufficient extraction. Too long a time will increase energy consumption and the expense of the extraction process [20]. The effect of time (10, 20, 30, 40, 50 and 60 min) on the extraction efficiency was investigated and the other extraction parameters were kept constant including water content 30%, extraction temperature 60 °C, and liquid–solid ratio 40 mL/g. The results are displayed in Figure 2e. It can be seen that the extraction efficiency was first increased and then decreased, and the maximum extraction efficiency (27.22 ± 0.31 mg/g) was obtained when the time was 40 min. This trend may be attributed to the fact that prolonged extraction time facilitated more sufficient contact between the solvent and TB powder, leading to more complete extraction. However, prolonged exposure to high temperatures may cause the degradation of flavonoid compounds; therefore, excessively long extraction times result in a slight decrease in the extraction yield [28]. Thus, 40 min was selected as the optimum time for the extraction.

#### 2.1.6. Comparison of Different Extraction Methods

Most of the reported methods for extracting flavonoids from TB used organic solvents. For instance, Zhang et al. [29] optimized the reflux extraction process of flavonoids from TB using 75% ethanol as the solvent. The highest extraction efficiency (9.01 mg/g) was achieved after 60 min of extraction under the condition of a liquid–solid ratio of 1:40 g/mL, which was lower than the 27.22 ± 0.31 mg/g of this study. In addition, ionic liquids, as green solvents, have also been used to extract flavonoids. For example, a 1.4 mol/L ionic liquid ([C_4_mim]Br) aqueous solution was used to extract flavonoids from TB at 35 °C for 45 min, achieving a high extraction efficiency of 41.17 mg/g, and it was higher than that of traditional organic solvents (70% ethanol, ethanol, and methanol) [5]. However, the cytotoxicity of ionic liquids cannot be ignored, and their cost is relatively high. Another relatively safe and inexpensive green solvent (DES) has also been used for the extraction of flavonoids from TB. Huang et al. [30] compared 13 different DESs for this purpose, and the results showed that choline chloride/glycerol had the highest extraction efficiency of 9.5 mg/g, which was lower than the that of DES-5 used in this study. It is evident that the method proposed in this study is efficient and worthy of further study and application.

### 2.2. Extraction Kinetic and Thermodynamic Analysis

**Figure 3:**
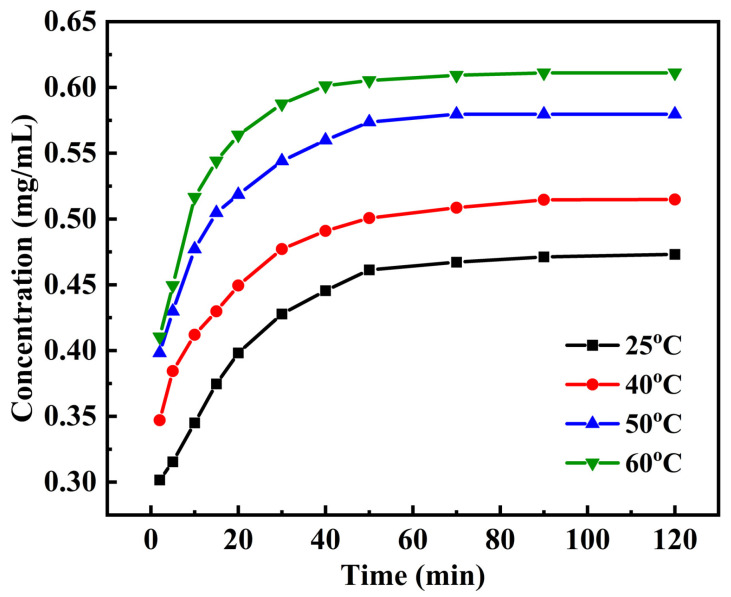
Influence of extraction time on the concentration of flavonoids (25 °C, 40 °C, 50 °C and 60 °C).

Extraction kinetic analysis plays a pivotal role in the regulation and prediction of extraction processes. To investigate this, 1.0 g of TB powder (40 mesh) was added into 40 mL DES-5 (choline chloride/glycolic acid, 1:2) with 30% water content, then the concentrations of flavonoids were measured at regular intervals to evaluate the extraction efficiency. As depicted in Figure 3, the extraction process consisted of two stages. In the initial stage, the concentration of flavonoids in the extract was significantly dependent on the extraction time, which can be regarded as the kinetic region of extraction [31]. As the extraction process progresses, the extraction rate decelerated, and the concentration of flavonoids eventually stabilized, indicating that the extraction process reached an equilibrium and entered into the second stage. Furthermore, the equilibrium concentration of flavonoids exhibited a positive correlation with temperature, suggesting that elevating the temperature from 25 °C to 60 °C facilitates the extraction of flavonoids from TB [32].

**Figure 4:**
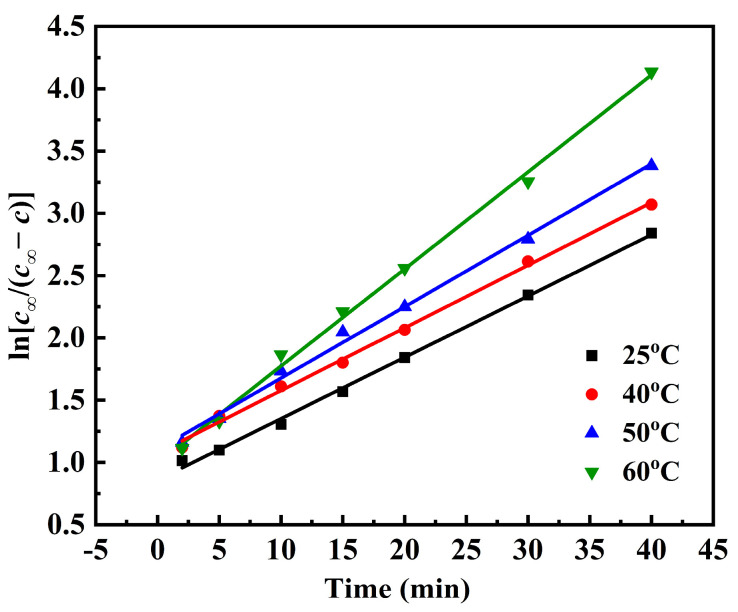
The relationship between $\ln\left(\frac{c_{\infty }}{c_{\infty }-c}\right)$ and extraction time (25 °C, 40 °C, 50 °C and 60 °C).

In addition, the correlation between $\ln\left(\frac{c_{\infty }}{c_{\infty }-c}\right)$ and extraction time (t) is visualized in Figure 4. The data exhibited a strong linear correlation, and the corresponding fitted linear equations are summarized in Table 1.

As detailed in Table 1, the coefficient of determination (R^2^) of all fitted linear equations was higher than 0.99, indicating that the extraction process fitted well with Fick’s second law [33]. Then, based on the fitted equation, the extraction rate constants (*k*) for flavonoids were determined to be 0.0493, 0.0503, 0.0573 and 0.0779 min^−1^ at 25 °C, 40 °C, 50 °C and 60 °C, respectively. The observed increase in *k* with rising temperatures suggests that elevated temperatures significantly accelerate the extraction process [34].

**Figure 5:**
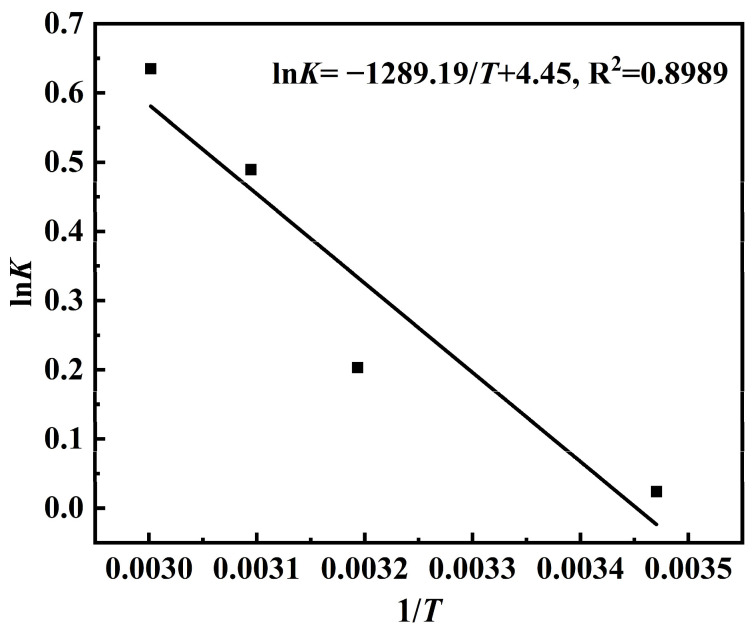
The relationship between ln*K* and 1/*T*.

To further explore the extraction mechanism, three thermodynamic parameters (Δ*G*, Δ*H* and Δ*S*) were determined by Equations (3) and (4). The relationship between ln*K* and 1/T is shown in Figure 5, while the calculated results are summarized in Table 2.

As presented in Table 2, the negative values of Δ*G* indicated that the extraction process was spontaneous. Furthermore, the value of Δ*G* decreased with an increase in temperature, suggesting that the extraction was more spontaneous at a higher temperature [35]. The positive value of Δ*H* indicated that the extraction process was endothermic [36], consistent with the observation that higher temperatures enhance extraction efficiency. Additionally, the positive value of Δ*S* suggested that the extraction process was irreversible [37].

### 2.3. Recovery of Flavonoids

#### 2.3.1. Screening of Macroporous Resins

**Figure 6:**
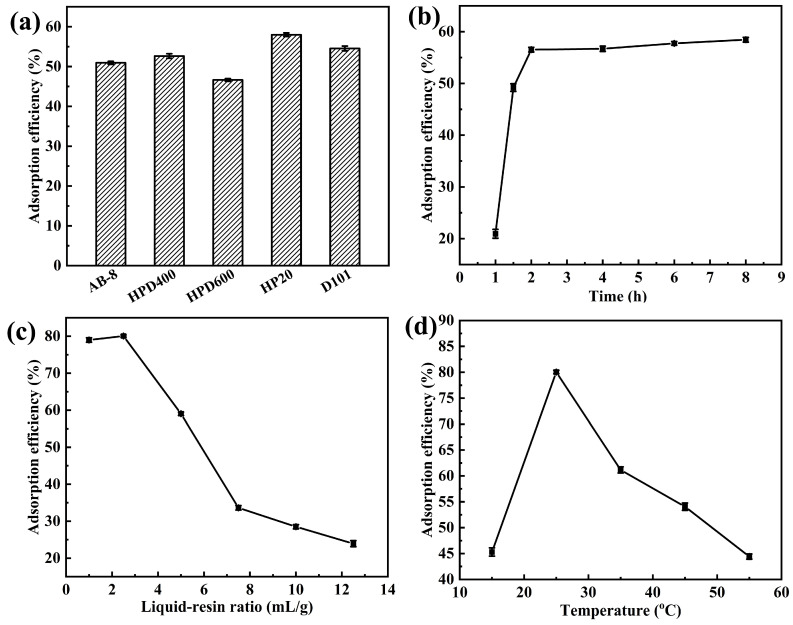
Effect of the (**a**) resin types, (**b**) adsorption time, (**c**) liquid–resin ratio and (**d**) adsorption temperature on the adsorption efficiency of flavonoids.

In general, the adsorption of resins is related to their properties, including pore size, surface area, and polarity. In order to select the optimal adsorbent for enriching flavonoids in TB extract, the adsorption efficiency of five different resins (AB-8, HPD400, HPD600, HP20, D101) was tested. According to the results in Figure 6a, HP20 (non-polar) had the highest adsorption efficiency, followed by D101 (non-polar), while the polar resin HPD600 showed the lowest adsorption efficiency. This is mainly because the flavonoids have relatively low polarity and are not suitable for resins with high polarity [38]. Moreover, HP20 had a higher adsorption efficiency due to its larger specific surface area (550–600 m^2^/g) compared to D101 (500–550 m^2^/g). Therefore, HP20 was selected for the following research.

#### 2.3.2. Effect of the Adsorption Time on Adsorption Efficiency

In this study, the adsorption was carried out at 25 °C with a liquid-to-resin ratio of 5 mL/g. The results are shown in Figure 6b.

In Figure 6b, the adsorption efficiency increased quickly till the adsorption stage arrived at 1.5 h, then the increase speed decreased. The rapid increased adsorption efficiency could be attributed to the rapid attachment of flavonoids to the active sites on the macroporous and mesoporous resins, resulting in smaller diffusion hindrance and faster moving speed. After 1.5 h, the adsorption was mainly carried out by the active sites on the surface of the resin micropores, and the diffusion of flavonoids experienced more hindrance, resulting in a corresponding decrease in adsorption speed [2]. Furthermore, when the adsorption time was 2 h, the adsorption efficiency tended to be constant. This indicated that 2 h was enough for flavonoids to absorb into the resins, and the adsorption process was of a rapid equilibrium type, which contributed to industrial production [39].

#### 2.3.3. Effect of the Liquid–Resin Ratio on Adsorption Efficiency

The effect of the liquid–resin ratio on adsorption efficiency is shown in Figure 6c. It was observed that as the liquid–resin ratio decreased, adsorption efficiency increased, and remained basically unchanged until the liquid–resin ratio decreased to 2.5. This was because the adsorption only occurred at the sites with high activity in the macroporous region in the initial stage, and then gradually shifted to the low-activity sites. When the liquid–resin ratio was small, the amount of flavonoids in the adsorption solution was relatively low, and they were only adsorbed at highly active sites, resulting in a high adsorption efficiency. As the volume of the adsorption solution increased, the relative amount of flavonoids increased. In addition to highly active sites, there would be more relatively weak active sites adsorbing flavonoids until the adsorption equilibrium was reached. When the volume increased excessively, the amount of flavonoids in the solution increased, but there were no more active sites on the resins to adsorb and the adsorption amount no longer increased, resulting in a decrease in adsorption efficiency [40].

#### 2.3.4. Effect of the Adsorption Temperature on Adsorption Efficiency

The effect of the adsorption temperature on adsorption efficiency is shown in Figure 6d. The adsorption efficiency increased from 15 °C to 25 °C, but decreased with an increase in temperature from 25 °C to 55 °C. Some research has reported that the adsorption process of flavonoids is an exothermic process, and low temperature is conducive to the adsorption process [41,42,43]. In this experimental result, the adsorption efficiency at 15 °C was lower than that at 25 °C, which may be due to the slower molecular motion at lower temperatures, and the adsorption equilibrium was not reached within 2 h in this experiment, resulting in a lower adsorption efficiency. For the above reason, 25 °C was selected as the optimal temperature.

Finally, the adsorption efficiency of flavonoids on HP20 resins under the optimal conditions (time 2 h, liquid–resin ratio 2.5 mL/g, temperature 25 °C) could reach 80.14%.

#### 2.3.5. Screening of Eluent Solvent

**Figure 7:**
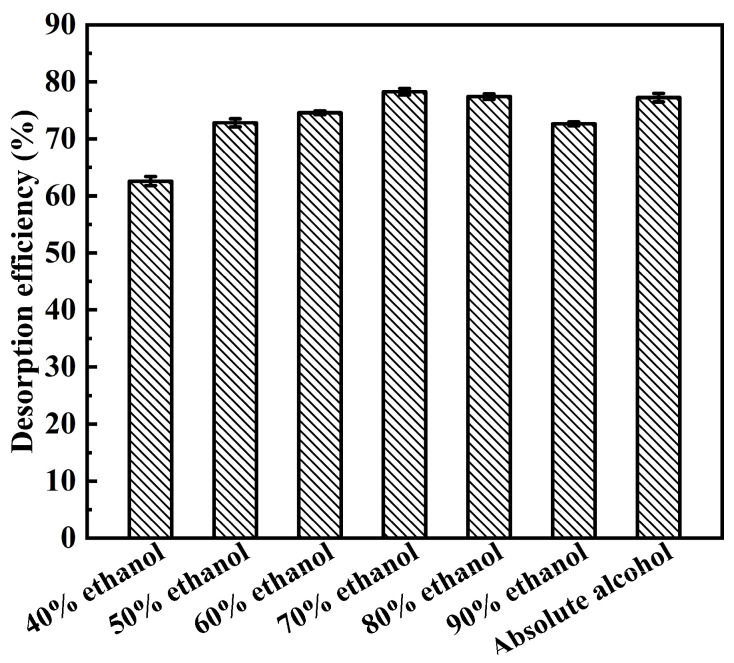
Effect of the eluent types on the desorption efficiency of flavonoids.

Desorption was the final step in recovering the target compounds. In traditional solvent extraction, many eluents have been used to recover flavonoids from macroporous resins. Among these eluents, ethanol has the characteristics of being effective and recyclable [44]. The desorption was related to the polarity of the eluent and depends on the solubility of flavonoids [45]. In this study, the eluent was selected from ethanol aqueous solutions of different concentrations. In Figure 7, as the ethanol concentration increases from 40% to 70%, the desorption efficiency increases to 78.27 ± 0.58%, and then gradually decreases as the ethanol concentration continues to increase. The concentration of 70% ethanol was sufficient for the desorption of the flavonoids from the adsorbents. This may be the result of the interaction between flavonoids and resins, as well as their dissolution in solvents. In this study, 70% ethanol provided the optimal environment, ensuring high solubility of the flavonoids in the solvent and effectively reducing its adsorption affinity on non-polar resin (HP20), thereby achieving efficient elution. In addition, the presence of impurities that were insoluble or difficult to dissolve in ethanol could also affect the desorption efficiency by covering the resin surface [46].

## 3. Materials and Methods

### 3.1. Materials and Instruments

Choline chloride, glycolic acid, urea, glycerol, 1,3-propanediol, levulinic acid, methanol and ethanol were of analytical reagent grade and purchased from Kelong chemical reagent factory (Chengdu, China); D101 and AB-8 macroporous resins were purchased from Macklin Biochemical Technology Co., Ltd. (Shanghai, China); HP20 macroporous resins were purchased from Solarbio Technology Co., Ltd. (Beijing, China), and their particle size was 0.3–1.25 mm; HPD400 and HPD600 macroporous resins were purchased from Yuanye Bio-Technology Co., Ltd. (Shanghai, China). All the macroporous resins were pretreated with absolute ethanol, 5% HCl and 5% NaOH in turn before use according to the manual. Standard sample of rutin (purity above 98.0%) was purchased from Alfa-biotech company (Chengdu, China); Tartary buckwheat (*Fagopyrum tataricum* Gaertn.) was collected from Xichang city in Sichuan province of China. It was ground into 40 mesh powder and kept in dark vacuum desiccator at room temperature for further use.

TU-1810DPC UV-Vis spectrophotometer (Beijing Persee General Instrument Co., Ltd., Beijing, China) was used for the determination of flavonoids. DF-101S homothermal system (Yuhua Instrument Co. Ltd., Gongyi, China) was used for synthesis and extraction experiments. SHA-C constant temperature shaker (Jiangsu Jinyi Instrument Technology Co., Ltd., Changzhou, China) was used for adsorption and desorption.

### 3.2. Preparation of DES

In this study, choline chloride was chosen as the HBA; glycerol, 1,3-propanediol, urea, levulinic acid and glycolic acid were chosen as HBDs. Referring to previous study [5], 5 DESs (see Table 3) were prepared by combining the corresponding HBA and HBD at predetermined molar ratios, followed by magnetic stirring at 85 °C for 3 h. After preparation, the DESs were cooled, sealed, and stored at ambient temperature for 72 h to verify their stability against crystallization prior to experimental application.

### 3.3. Extraction Procedure

TB powder (40 mesh) was mixed with DES aqueous solution at a liquid-to-solid ratio of 40 mL/g, and the extraction was conducted under magnetic stirring at 50 °C for 30 min. After extraction, the samples were centrifuged at 8000 rpm for 5 min, and the flavonoid content in the supernatant was determined.

#### 3.3.1. Quantification of TB Flavonoids

Flavonoid concentration was determined via UV-Vis spectrophotometry using rutin as the standard. The regression equation was *y* = 24.7321*x* + 0.0168, where *x* is the absorbance at 359 nm and *y* is the concentration of flavonoids (mg/mL), and the calibration curve exhibited good linearity over the concentration range of 0.005–0.035 mg/mL (R^2^ = 0.9996). Based on the determined concentration and the volume of the extract, the total mass of flavonoids was calculated. Subsequently, the extraction efficiency (*E*, mg/g) was calculated by Equation (1):

(1) $$E=\frac{m_{1}}{m}$$

where *m*_1_ (mg) is the mass of the obtained flavonoids, and *m* (g) represents the mass of TB powder.

#### 3.3.2. Investigation on Extraction Conditions

To systematically optimize the extraction conditions, multiple factors were evaluated, including the type of DES (DES-1–DES-5), the water content (10–50%, *v*/*v*), liquid–solid ratio of extractant and TB powder (20–60 mL/g), extraction temperature (30–70 °C) and extraction time (10–60 min).

#### 3.3.3. Extraction Kinetic and Thermodynamic Analysis

It is widely recognized that the extraction of bioactive compounds from medicinal plants involves solvent permeation, solute dissolution, and diffusion. Among these steps, solute diffusion is considered the rate-limiting factor, driven by the concentration gradient between the solid and liquid phases [33]. In addition, previous research has shown the extraction of active ingredients from natural products as an unstable diffusion process [47]. The study of dynamic models is crucial for optimizing extraction conditions and predicting extraction processes, and the establishment and modeling of the kinetic mechanism is useful for large-scale extraction and production [48]. Consequently, based on Fick’s second law and previous study [49], Equation (2) was used in the study to investigate the extraction kinetics.

(2) $$\ln\left(\frac{c_{\infty }}{c_{\infty }-c}\right)=kt+\ln\left(\frac{c_{\infty }}{c_{\infty }-c_{0}}\right)$$

where $c_{0}$, c and $c_{\infty }$ (mg/mL) are the concentration of flavonoids in the extract at the beginning of extraction, during the extraction process and at the extraction equilibrium, respectively.

Furthermore, three thermodynamic parameters, including Gibbs free energy change (Δ*G*), enthalpy change (∆*H*) and entropy change (∆*S*), were calculated by Equations (3) and (4), as described in reference [50], to further elucidate the extraction mechanism.

(3) $$\ln K=-\frac{\Delta H}{R}\frac{1}{T}+\frac{\Delta S}{R}$$

(4) ΔG=ΔH−TΔS

where R is the molar gas constant, 8.314 J/(mol·K); *T* (K) is the temperature; and *K* is the transfer constant of flavonoids, which can be obtained by the concentration in the extract (*C_t_*) and TB powder (*C_s_*) at the extraction equilibrium. Due to the difficulty in directly determining the *C_s_* in this study, we calculated it by subtracting *C_t_* from the initial total flavonoid content in the raw material (*C_s_*_,0_). Theoretically, *C_s_*_,0_ equals the sum of concentrations obtained from infinite extraction cycles [36]. So, DES-5 (choline chloride–glycolic acid, 1:2) with 30% water was used as the extraction solvent, with a liquid-to-solid ratio of 40 mL/g, and the extraction was repeated multiple times at 60 °C for 60 min. The flavonoid concentration of the sixth extraction solution was negligible, indicating that the flavonoids in the TB powder were nearly completely exhausted after 5 cycles. Therefore, the sum of the results from the first 5 extractions was taken as the value of *C_s_*_,0_, and it was determined to be 0.9349 mg/mL.

### 3.4. Recovery of Flavonoids

The separation of extractant and flavonoids after the extraction always needs to be considered. However, distillation is unsuitable for solvent recovery due to the negligible vapor pressure of the DES [51].

In this study, the commonly used resin was selected to recover flavonoids, and the adsorption and desorption experiment followed a previously established protocol, with appropriate adjustments [52]. Briefly, a specific amount of activated macroporous resin was mixed with the extracts; then the mixture was incubated in a temperature-controlled water-bath oscillator at 100 rpm for a specified time. After the adsorption process was complete, the resin was separated from the supernatant by filtration. Then the amount of un-adsorbed flavonoids was quantified using the calibration curve in Section 3.3.1, and the adsorption efficiency (*E*, %) was calculated using Equation (5) [53].

(5) $$E=\frac{C_{0}-C_{1}}{C_{0}}\times 100$$

where $C_{0}$, $C_{1}$ are the concentrations of flavonoids before and after adsorption, respectively.

In this study, five macroporous resins previously reported for flavonoid adsorption were selected, namely AB-8 (weakly polar), HPD400 (moderately polar), HPD600 (polar), HP20 (non-polar), and D101 (non-polar) [54,55,56,57,58]. Firstly, suitable resins were screened, and then a systematic investigation of the adsorption conditions was performed, mainly including adsorption time (1, 1.5, 2, 4, 6 and 8 h), liquid–resin ratio (1, 2.5, 5, 7.5, 10 and 12.5 mL/g), and adsorption temperature (15 °C, 25 °C, 35 °C, 45 °C and 55 °C).

After that, the flavonoid-adsorbed resins were eluted with eluent by stirring (100 rpm) at 25 °C for 2 h. Finally, the eluent was collected from resins by filtration for the determination of flavonoid content, and the desorption efficiency (*D*, %) can be obtained by the following Equation (6) [59].

(6) $$D=\frac{C_{D}V_{D}}{{\left(C_{0}-C_{1}\right)V}_{A}}\times 100$$

where $C_{0}$ and $C_{1}$ are the concentrations of flavonoids before and after adsorption, respectively; $C_{D}$ is the concentrations of flavonoids in eluent after the desorption; and $V_{A}$ and $V_{D}$ are the volume of adsorption and desorption solution, respectively.

### 3.5. Statistical Analysis

Statistical analysis was performed with Microsoft Excel 2024. All experiments were performed in triplicate, and the results were expressed as mean ± standard deviation (SD). Bar charts, line graphs, and correlation analysis were generated using OriginPro 2021.

## 4. Conclusions

In the present study, an effective extraction method using DESs was successfully applied to extract flavonoids from Tartary buckwheat. Among the tested DESs, DES-5 (choline chloride/glycolic acid, 1:2) was the good choice. The optimal extraction conditions (water content of 30%, liquid–solid ratio of 40 mL/g, extraction temperature of 60 °C and extraction time of 40 min) were assessed by single-factor experiments, and the extraction efficiency was 27.22 ± 0.31 mg/g. Furthermore, it was found that Fick’s second diffusion law was suitable to describe the extraction process in extraction kinetic and thermodynamic analysis. It was also found that this was an endothermic process, and the randomness of the system increased during the extraction process. Finally, flavonoids were recovered by adsorption with HP20 macroporous resins; the adsorption efficiency under the optimal conditions (time 2 h, liquid–resin ratio 2.5 mL/g, temperature 25 °C) could reach 80.14 ± 0.33%. And the concentration of 70% ethanol was sufficient for the desorption of the flavonoids from the adsorbents. Therefore, the developed method showed good performance in the extraction process of flavonoids from Tartary buckwheat, which contributed to the wide application of DESs in extraction, and also provided a theoretical foundation for their practical applications.

## Figures and Tables

**Table 1:** The fitted linear equations of flavonoids (25 °C, 40 °C, 50 °C and 60 °C).

Temperature (°C)	Fitted Equation	R^2^
25	*y* = 0.0493*x* + 0.8587	0.9977
40	*y* = 0.0503*x* + 1.0735	0.9970
50	*y* = 0.0573*x* + 1.1034	0.9958
60	*y* = 0.0779*x* + 0.9947	0.9969

**Table 2:** The extraction thermodynamic parameters.

Temperature (°C)	∆*G* (kJ/mol)	∆*H* (kJ/mol)	∆*S* [kJ/(mol·K)]
25	−0.06225	10.7183	0.037413
40	−0.99758		
50	−1.37171		
60	−1.74584		

**Table 3:** The list of the prepared DESs.

Abbreviation	HBA	HBD	Molar Ratio	Appearance at 25 °C
DES-1	choline chloride	glycerol	1:2	colorless transparent liquid
DES-2	choline chloride	1,3-propanediol	1:2	colorless transparent liquid
DES-3	choline chloride	urea	1:2	colorless transparent liquid
DES-4	choline chloride	levulinic acid	1:2	light yellow transparent liquid
DES-5	choline chloride	glycolic acid	1:2	colorless transparent liquid

## Data Availability

Data are contained within the article.
